# HSPB1 Regulates Autophagy and Apoptosis in Vascular Smooth Muscle Cells in Arteriosclerosis Obliterans

**DOI:** 10.1155/2022/3889419

**Published:** 2022-11-14

**Authors:** Keqin Chen, Changmiao Hou, Lei Xu, Hanwu Peng, Chaogui He, Jing Liu, Guoqing Wang, Shaoshuai Huang, Xiehong Liu

**Affiliations:** ^1^Department of Vascular Surgery, The First Hospital of Changsha, Changsha, Hunan, China; ^2^Hunan Provincial Key Laboratory of Emergency and Critical Care Metabonomics, Institute of Emergency Medicine, The First Affiliated Hospital of Hunan Normal University (Hunan Provincial People's Hospital), Changsha, Hunan, China; ^3^Hunan University of Traditional Chinese Medicine, Changsha, Hunan, China; ^4^Public Health Clinical Center, Xiangtan Central Hospital, Xiangtan, Hunan, China

## Abstract

**Objective:**

Small heat shock protein-1 (HSPB1) is a small heat shock protein that participates in many cellular processes and alleviates stress-induced cell injury. Autophagy protects cells from many types of stress and plays a key role in preventing stress in arteriosclerosis obliterans (ASO). However, the roles of HSPB1 in autophagy and apoptosis in the context of ASO pathogenesis remain unclear.

**Methods:**

In vivo and in vitro studies were used to determine whether HSPB1 is associated with ASO progression. The expression of HSPB1 was measured in normal and sclerotic blood vessels. The role of HSPB1 and its potential downstream signaling pathway were determined in VSMCs by overexpressing and silencing HSPB1.

**Results:**

A total of 91 ASO patients admitted to and treated at our hospital from Sep. 2020 to Sep. 2021 were selected, and plasma HSPB1 expression was assessed. We divided the patients with ASO into the grade I (*n* = 39), II (*n* = 29), III (*n* = 10), and IV (*n* = 13) groups according to Fontaine's classification. Plasma HSPB1 levels were markedly decreased in patients with grade III (*n* = 10) and IV (*n* = 13) ASO compared with patients with grade I ASO. Furthermore, HSPB1 expression was significantly decreased, and p62 and cleaved caspase-3 were increased in the sclerotic vasculature compared to the normal vasculature (*p* < 0.05). Overexpression of HSPB1 promoted apoptosis of VSMCs following ox-LDL treatment. Knockdown of HSPB1 led to a marked increase in the expression of LC3II and Beclin-1 in ox-LDL-stimulated VSMCs, whereas knockdown of HSPB1 attenuated these changes (*p* < 0.05). Importantly, overexpression of HSPB1 promoted the dephosphorylation of JNK in ox-LDL-stimulated VSMCs. Conversely, downregulation of HSPB1 induced the opposite change.

**Conclusion:**

Loss of HSPB1 promotes VSMC autophagy and inhibits VSMC apoptosis, which are associated with ASO. HSPB1 and its downstream signaling pathways could be potential therapeutic targets for ASO treatment.

## 1. Introduction

Arteriosclerosis obliterans (ASO) is one of the most common peripheral arteriosclerotic vascular diseases [1], but the etiology of ASO is not completely clear. Smoking, hyperlipidemia, diabetes, hypertension, and hyperhomocysteinemia were reported to be risk factors for ASO [2]. Currently, intravascular interventions and bypass surgery are used to slow the progression of ASO; however, the postoperative restenosis rate remains high, which seriously affects the prognosis of ASO [3].

An increasing number of studies have reported the important roles of apoptosis and autophagy in atherosclerosis [4, 5]. Studies have shown that apoptosis of VSMCs promotes the occurrence of atherosclerosis [6]. Apoptosis of VSMCs is closely related to the thinning of the fibrous cap and the rupture of plaques in advanced atherosclerosis. Furthermore, apoptosis of VSMCs leads to calcification of atherosclerotic plaques, medial dilation and degeneration, inflammation, and atherosclerotic stenosis [7, 8]. Studies have revealed that enhanced VSMC autophagy inhibits ox-LDL-induced foam cell formation [9, 10]. In addition, dysfunction of VSMC autophagy contributes to the development of atherosclerosis by leading to cell death and instability of atherosclerotic plaques [11].

Small heat shock protein B1 (HSPB1) is an important member of the small heat shock protein superfamily and is highly expressed in cardiac, smooth, and skeletal muscles [12]. It plays a protective role in the myocardium by participating in the regulation of apoptosis, maintaining the integrity of the cytoskeleton, and protecting cells from oxidative stress [13]. HSPB1 exerts antiapoptotic effects at multiple levels by interfering with the expression of the proapoptotic proteins Bax and Bid and isolating cytochrome c released by mitochondria, thus inhibiting the caspase cascade [14, 15]. Mutation of HSPB1 impairs the formation of SQSTMI/p62 bodies, resulting in a decrease in autophagic flux, while wild-type HSPB1 expression rescues autophagic flux in cells with HSPB1 knockout [16]. Overexpression of HSPB1 increases autophagic flux and inhibits apoptosis induced by H_2_O_2_ in rats with acute kidney injury [17]. However, it is unclear how HSPB1 exerts an antiatherosclerotic effect by regulating apoptosis and autophagy.

In this study, we sought to clarify the mechanism of ASO from the perspective of HSPB1-mediated regulation of VSEC apoptosis and autophagy. We showed that HSPB1 could serve as a key molecule in balancing VSEC apoptosis and autophagy. Our study further elucidates the pathophysiology of ASO and may provide a new target for ASO intervention.

## 2. Materials and Methods

### 2.1. Human Blood Specimens

We performed this work after obtaining approval from the Institutional Ethics Committee of the First Hospital of Changsha. In our study, 91 patients of both sexes aged more than 58 years were recruited from the First Hospital of Changsha, Changsha, Hunan, China, after they provided written informed consent. Lower extremity ASO was diagnosed by medical history and physical examination. The ankle/brachial index and pulse wave velocity are widely used for the diagnosis of ASO. The main complaints of all ASO patients were chronic limb ischemia, intermittent claudication, resting pain, or unhealed ischemic ulcer. ASO was classified as grade I (*n* = 39), II (*n* = 29), III (*n* = 10), or IV (*n* = 13) according to the criteria of Fontaine. Plasma was isolated from blood samples from each patient (centrifuged at 3,000 rpm for 10 min). Vascular smooth muscle cells were collected from patients with sclerotic blood vessels, while smooth muscle cells from amputees were used as controls. Plasma and intimal samples were stored at -80°C for further analysis.

### 2.2. Isolation of Human Vascular Smooth Muscle Cells (HVSMCs)

HVSMCs were isolated as previously described [18]. Briefly, surgically resected sclerosed vascular tissues were immersed in Hank's balanced salt solution (HBSS) containing penicillin and streptomycin (5 × 10^5^ U. L^−1^) and incubated at 4°C for 30 min. The tissue surrounding the sclerosed vascular tissue mass and the denatured vascular tissue were removed by electrocauterization, and the blood and denatured tissue were washed away by HPSS. The vessel was then cut longitudinally, the intimal endothelial cells were gently scraped away, and the inside and outside of the vessel were washed several times with HPSS. The tissues were then minced into small pieces (0.2 cm^3^) and digested with trypsin (4 mg/ml) and collagenase P (1.0 mg/ml) at 37°C for 45 min. Then, the VSMCs were recovered by centrifugation before being resuspended in growth medium supplemented with medium 199 (×1), 10% FBS, and 100 U/ml penicillin and streptomycin. The VSMCs were then incubated at 37°C and 5% CO_2_ in a humidified incubator, and the culture medium was changed after 24 hr. Two-thirds of the culture medium was changed every 3 days, and the cells were subcultured when the cell density reached 95%.

### 2.3. Cell Culture

VSMCs were cultured in Dulbecco's modified Eagle's medium supplemented with 10% fetal bovine serum and 100 U/ml penicillin/100 *μ*g/ml streptomycin in a humidified atmosphere containing 5% CO_2_ at 37°C. The cells were incubated for 24 hr, transiently transfected with pCMV-Cmyc-HSPB1 or HSPB1 small interfering RNA (siRNA) for 48 hr, and then treated with 60 *μ*g/ml ox-LDL for 24 hr. After treatment, the cells were harvested for analysis.

### 2.4. Estimation of HSPB1 Levels by ELISA

According to the manufacturer's protocol, the Human HSP27 ELISA Kit (directory #ek1244, Multi Sciences Biotechnology, Co., Hangzhou, China) was used to measure the plasma level of HSPB1. In brief, an enzyme-precoated microplate was washed with washing buffer for 30 seconds. Diluted sample and a horseradish peroxidase- (HRP-) labeled antibody (sample : antibody = 100 : 1) were added to the microplate and incubated for 2 hr at 37°C with a vibration at a speed of 300 rpm/min. Then, the plate was washed 6 times. The chromogenic substrate TMB was added, and the microplate was incubated for 30 min at room temperature in the dark. Stop solution was added to stop the reaction. Importantly, it was ensured that the addition reagents were thoroughly mixed. The absorbance was measured using a microplate spectrophotometer at 450 nm, and the wavelength correction was set to 630 nm. The intra- and interassay coefficients of variation for HSPB1 ELISA were 7.8% and 9.3%, respectively.

### 2.5. Western Blot Analysis

Vascular tissues were ground under liquid nitrogen and then suspended in lysis buffer containing complete protease inhibitor. Subsequently, the proteins were separated by 10% SDS–PAGE and transferred to PVDF membranes. Following blocking with 5% skimmed milk for 1.5 h, the membranes were incubated with primary antibodies against *α*-SMA (cat. no. AF1032; Affinity Biosciences), HSPB1 (cat. no. ADI-SPA-801-F; Enzo Life Sciences, Farmingdale, NY), p62 (cat. no. #5114S; Cell Signaling Technology), and cleaved-caspase 3 (cat. no. #9662S; Cell Signaling Technology) overnight at 4°C. After washing in TBST, the membranes were incubated with the corresponding secondary antibody (cat. no. 7074; Cell Signaling Technology) for 1.5 h. Finally, the immunoblot signals were visualized with a ChemiDoc™ Imaging System (BLM Biotechnology Co., Ltd.). The band density was quantified using the ImageJ software (NIH, Bethesda, MD).

Total protein was extracted from VSMCs using RIPA lysis buffer following treatment, and the protein concentration was quantified using a BCA Protein Assay Kit. Western blotting was performed as described above. We used antibodies against PCNA (cat. no. AF0239; Affinity Biosciences), Ki67 (cat. no. AF0198; Affinity Biosciences), Bcl-2 (cat. no. PRS3335; Sigma-Aldrich), Bax (cat. no. SAB4504350; Sigma-Aldrich), LC3 (cat. no. #12741S; Cell Signaling Technology), Beclin-1 (cat. no. ab62557; Abcam Pharmatech), phospho-SAPK/JNK (Thr183/Tyr185) (cat. no. #4668; Cell Signaling), SAPK/JNK (cat. no. #9252; Cell Signaling), and *β*-actin (cat. no. AF7018; Affinity Biosciences) to assess protein expression.

### 2.6. Analysis of Apoptosis by Annexin V/Propidium Iodide (PI) Staining

VSMCs (1 × 10^5^ cells/well) were seeded in 6-well plates for 24 hr. Then, the cells were transfected with plasmids and siRNAs using Lipofectamine® 2000 (Life Technologies) for 48 hr and treated with 60 *μ*g/ml ox-LDL for another 24 hr. Subsequently, cell apoptosis was analyze with the annexin V-FITC Apoptosis Detection Kit (MilliporeSigma) according to the manufacturer's instructions. Briefly, after washing the cells twice with PBS, they were resuspended in annexin V-binding buffer and incubated with annexin V-FITC and PI for 15 min in darkness. Analysis was then performed using the FACSCalibur and BD CellQuest™ Pro software version 6.0 (both BD Biosciences). The apoptosis rate was calculated according as follows: the number of early apoptotic cells plus the number of late apoptotic cells/the total number of cells.

### 2.7. Transmission Electron Microscopy (TEM)

VSMCs were collected, fixed with 5% glutaraldehyde for 2 hr, and postfixed in 1% osmic acid for 2 hr. The cells were then dehydrated in an alcohol gradient and acetone, embedded in EPON at 37°C for 12 hr, and solidified at 60°C for 24 hr. The embedding blocks were sliced into 70 nm thick ultrathin sections on a microtome (Leica, Germany). The ultrathin sections were sequentially stained with both 3% uranyl acetate and lead citrate. Images were captured and analyzed with a transmission electron microscope (Hitachi TEM system HC-1, Japan).

### 2.8. Data Analysis

Continuous and categorical variables representing baseline characteristics are presented as the median (interquartile range) and *n* (%), and significance was analyzed by *χ*^2^ test or Fisher's exact test where appropriate. A two-sided *P* value of 0.05 was considered significant. Each experiment was repeated three times. For Western blotting, one representative image is shown. The results are presented as means ± standard deviations. Statistical significance was analyzed using one-way analysis of variance and Tukey's multiple comparison tests. Data analysis was performed with GraphPad Prism (La Jolla, CA).

## 3. Results

### 3.1. Clinical Profiles of Patients across the Groups

From Sep. 2020 to Sep. 2021, a total of 91 patients were enrolled in the clinical study (Table 1). The participants were divided into four groups, i.e., the grade I (*n* = 39), II (*n* = 29), III (*n* = 10), and IV (*n* = 13) groups, according to Fontaine's classification. None of the following parameters differed among the four groups: sex; age; smoking status; drinking status; hypertension, hyperlipidemia, or diabetes mellitus incidence; and TG level. The levels of FPG (*p* = 0.0005), TC (*p* = 0.022), and LDL-C (*p* = 0.045) were markedly increased, and HDL-C levels (*p* = 0.006) were decreased in grade IV ASO patients compared with grade I ASO patients (Table 1).

### 3.2. Loss of HSPB1 Is Associated with ASO Progression and VSMC Apoptosis

**Figure 1:**
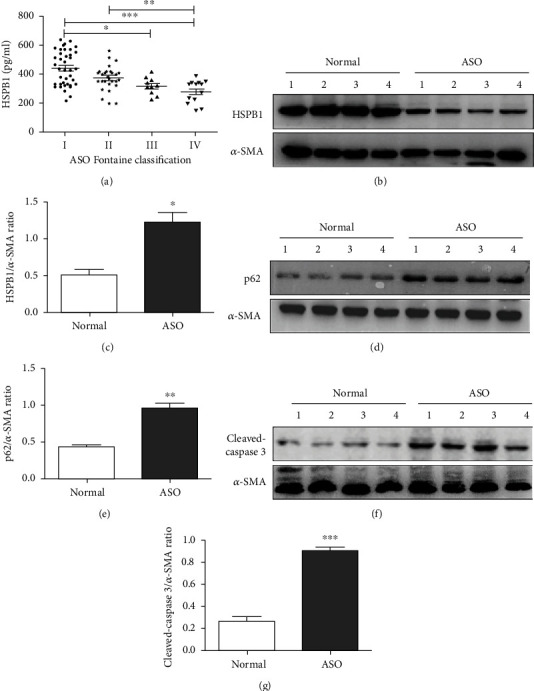
Expression of HSPB1 in ASO patients. (a) ELISA was used to quantify the plasma levels of HSPB1. The absorbance was measured using a microplate spectrophotometer at 450 nm with wavelength correction set to 630 nm. The error bars are the SDs of 3 replicates. ^∗^*p* < 0.05, ^∗∗^*p* < 0.01, and ^∗∗∗^*p* < 0.001. (b–g) Sclerotic intima was collected from four patients clinically diagnosed with grade III ASO, and normal intima obtained from amputation patients was used as a control. The tissues were homogenized in RIPA buffer containing complete protease inhibitor, and then, Western blot analysis was performed to measure the protein expression of (b) HSPB1, (d) p62, and (f) cleaved caspase-3. Densitometry was used to determine the fold change in the expression of (c) HSPB1, (e) p62, and (g) cleaved caspase-3 relative to the expression of *β*-actin (*n* = 6 for each group; ^∗^*p* < 0.05, ^∗∗^*p* < 0.01, and ^∗∗∗^*p* < 0.001).

It is well documented that a low level of HSPB1 may be a potential biological indicator of atherosclerosis [17]. Therefore, we first determined whether the plasma level of HSPB1 was changed in patients with ASO. The ELISA results showed that HSPB1 levels in the grade II and III groups were obviously decreased compared to those in the grade I group (Figure 1(a)). Subsequently, we assessed the HSPB1 protein level in the vascular endothelial lesions of ASO patients. As shown in Figures 1(b) and 1(c), the expression of HSPB1 was significantly decreased in the ASO group compared to the control group. These results suggest that a lower level of HSPB1 within the vascular intima was highly associated with the progression of ASO.

Apoptosis and autophagy influence the development and progression of atherosclerosis [19]. Of note, vascular smooth muscle cells (VSMCs) play an important role in foam cell formation, a hallmark of ASO [20]. We evaluated the expression of cleaved caspase-3 and the autophagy repressor p62 in VSMCs. Our results showed that p62 (Figures 1(d) and 1(e)) and cleaved caspase-3 (Figures 1(f) and 1(g)) levels were significantly increased in the intima of ASO patients compared to the control intima. These results suggest that apoptosis and autophagy are significantly increased in VSMCs in the intima of ASO patients. Taken together, these results indicate that HSPB1 expression is associated with VSMC apoptosis and autophagy.

### 3.3. The Effect of HSPB1 on the Proliferation and Apoptosis of ox-LDL-Stimulated VSMCs

**Figure 2:**
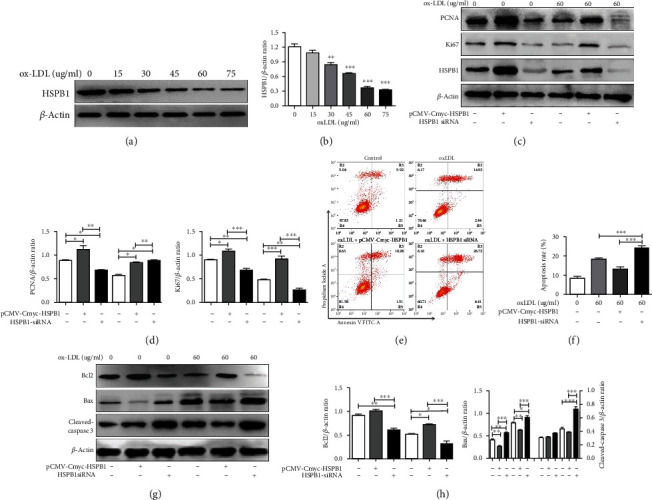
HSPB1 regulates apoptosis in VSMCs. (a) VSMCs were treated with 0, 15, 30, 45, 60, or 75 *μ*g/ml ox-LDL for 24 hr, and then, HSPB1 protein expression was measured by Western blotting. (b) Densitometry was used to calculate the fold change in the expression of HSPB1 relative to the expression of *β*-actin. (c–h) VSMCs were treated with pCMV-Cmyc-HSPB1 and HSPB1-siRNA for 48 hr, followed by 60 *μ*g/ml ox-LDL for 24 hr. (c) Cell lysates were harvested to examine the protein levels of PCNA, Ki67, and *β*-actin by Western blotting. (d) Densitometry was used to determine the fold change in the expression of PCNA and Ki67 relative to the expression of *β*-actin (*n* = 6 for each group; ^∗^*p* < 0.05, ^∗∗^*p* < 0.01, and ^∗∗∗^*p* < 0.001). (e) Representative flow cytometric plots are presented. (f) The data are presented as means ± standard deviations (*n* = 6). (g) Western blotting was performed as described above, and the protein levels of Bcl-2, Bax, cleaved caspase-3, and *β*-actin were measured. (h) The levels of these proteins were quantitated by densitometric analysis using ImageJ and normalized to the level of *β*-actin. The results are presented as the fold changes in expression compared with those in the control group (*n* = 6 for each group; ^∗^*p* < 0.05, ^∗∗^*p* < 0.01, and ^∗∗∗^*p* < 0.001).

To explore the function of HSPB1 in ASO progression, we investigated the effects of HSPB1 on the proliferation and apoptosis of ox-LDL-stimulated VSMCs. We first examined the effect of ox-LDL on HSPB1 expression in VSMCs by Western blotting. As shown, ox-LDL obviously decreased HSPB1 expression in VSMCs (Figures 2(a) and 2(b)). HSPB1 was then overexpressed or downregulated in VSMCs, and the cells were treated with 60 *μ*g/ml ox-LDL for 24 hr. These results clearly indicated that overexpression of HSPB1 upregulated the expression of proliferative markers (PCNA and Ki-67). In contrast, an apparent decrease in PCNA and Ki-67 expression was observed in VSMCs following HSPB1 downregulation (Figures 2(c) and 2(d)). Furthermore, flow cytometry showed that the percentage of apoptotic VSMCs was markedly decreased markedly when HSPB1 was overexpressed, while knockdown of endogenous HSPB1 using siRNA decreased the percentage of apoptotic cells (Figures 2(e) and 2(f)). Our results suggest that HSPB1 is required to protect VSMCs against ox-LDL-induced apoptosis.

Recently, a study showed that HSPB1 can alleviate oxidative stress-induced endothelial cell apoptosis by upregulating Bcl-2 and downregulating Bax [21]. As presented in Figures 2(g) and 2(h), caspase-3 activity and the expression of the proapoptotic protein Bax were markedly increased and the expression of the antiapoptotic protein Bcl-2 was notably reduced in VSMCs with HSPB1 knockdown compared to negative control VSMCs. In summary, these results suggest that HSPB1 deficiency inhibits the proliferation and promotes the apoptosis of ox-LDL-stimulated VSMCs.

### 3.4. HSPB1 Promotes Beclin-1-Dependent Autophagy

**Figure 3:**
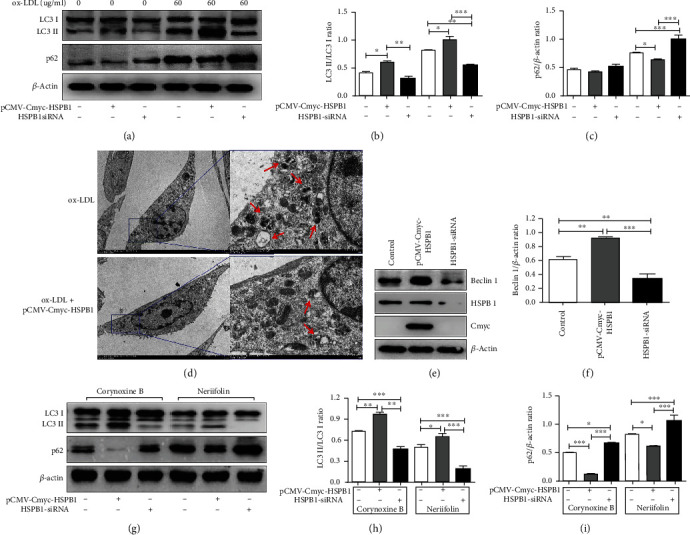
Tangeretin triggers autophagy in VSMCs. (a–c) VSMCs were treated with pCMV-Cmyc-HSPB1 and HSPB1-siRNA for 48 hr, followed by *μ*g/ml ox-LDL for 24 hr. (a) The levels of LC3, p62, and *β*-actin were examined. (b) Levels of LC3-II relative to those of LC3-I. (c) Levels of p62 relative to those of *β*-actin. (d) VSMCs were treated with pCMV-Cmyc-HSPB1 for 48 hr, followed by 60 *μ*g/ml ox-LDL for 24 hr, and then, TEM was used to observe autophagosomes. Scale bar, 5 *μ*m (×1.2 k, left) and 1 *μ*m (×6.0 k, right). (e, f) VSMCs were treated with pCMV-Cmyc-HSPB1 and HSPB1-siRNA for 48 hr. (e) Cell lysates were harvested to examine the protein levels of Beclin-1, HSPB1, Cmyc, and *β*-actin by Western blotting. (f) Densitometry was used to determine the fold change in the expression of Beclin-1 relative to the expression of *β*-actin. (g–i) VSMCs were treated with pCMV-Cmyc-HSPB1 and HSPB1-siRNA for 48 hr. Then, they were pretreated with 100 *μ*M corynoxine B and 1 *μ*M neriifolin for 2 h before being treated with 60 *μ*g/ml ox-LDL. (g) Cell lysates were harvested to examine the protein levels of LC3, p62, and *β*-actin by Western blotting. (h) Levels of LC3-II relative to those of LC3-I. (i) Levels of p62 relative to those of *β*-actin (*n* = 6 for each group; ^∗^*p* < 0.05, ^∗∗^*p* < 0.01, and ^∗∗∗^*p* < 0.001).

Recently, some studies have revealed that HSPB1 participates in the regulation of autophagy [22], and our above data also suggest that the expression of HSPB1 is negatively correlated with that of p62 in injured VSMCs in patients with ASO. Therefore, we sought to determine whether HSPB1 is responsible for the regulation of autophagy in VSMCs exposed to ox-LDL. Increased expression of HSPB1 markedly upregulated the expression of LC3II and decreased the expression of p62. In addition, knockdown of HSPB1 decreased the expression of LC3II and increased p62 expression (Figures 3(a)–3(c)). TEM showed that autophagosomes were more abundant in VSMCs in the ox-LDL groups than in those in the ox-LDL+ pCMV-Cmyc-HSPB1 group (Figure 3(d)). Taken together, these data suggest that HSPB1 has a regulatory effect on VSMC autophagy.

Beclin-1 is a critical autophagy-promoting gene that regulates the death and survival of VSMCs [23]. To clarify whether HSPB1 enhances VSMC autophagy through Beclin-1, we assessed the effect of changes in HSPB1 levels on Beclin-1 expression. The results showed that the expression of Beclin-1 was upregulated in the HSPB1 overexpression group but significantly decreased in the HSPB1 knockdown group compared to the control group (Figures 3(e) and 3(f)). In addition, to elucidate the relationship between HSPB1 and Beclin-1 in VSMCs treated with ox-LDL, we used an agonist (corynoxine B) and an inhibitor (neriifolin) of Beclin-1 to further examine the effects of HSPB1 on Beclin-1-dependent autophagy. As demonstrated in Figures 3(g)–3(i), the increase in p62 expression and the decrease in LC3II expression following HSPB1 knockdown in ox-LDL-treated cells were markedly attenuated by corynoxine B. In contrast, neriifolin reversed the increase in the expression of LC3II and the decrease in the expression of p62 caused by HSPB1 overexpression. These results clearly indicate that Beclin-1 serves as a target of HSPB1 in VSMCs.

### 3.5. JNK Participates in HSPB1-Mediated Autophagy in ox-LDL-Stimulated VSMCs

**Figure 4:**
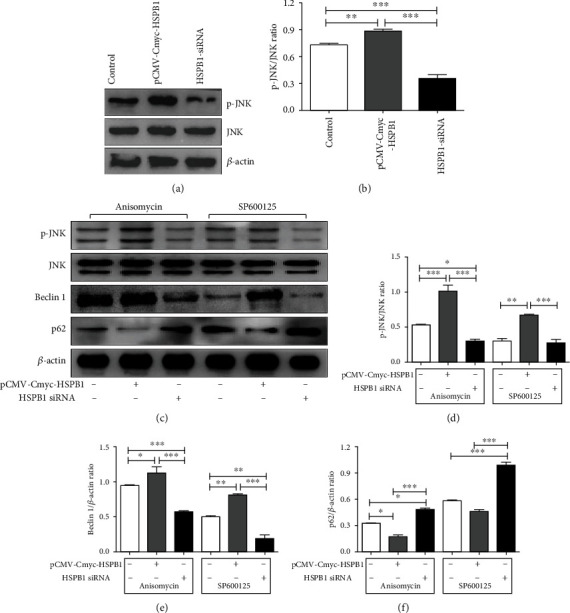
HSPB1 activates JNK and disrupts Beclin-1-associated autophagy. VSMCs were treated with pCMV-Cmyc-HSPB1 and HSPB1-siRNA for 48 hr, and (a) the levels of p-JNK, JNK, and *β*-actin were examined. (b) Levels of p-JNK relative to those of JNK. (c–f) Cells were pretreated with 1 mM anisomycin and 5 *μ*M SP600125 for 30 min before being treated with 60 *μ*g/ml ox-LDL. (c) Cell lysates were harvested to examine the protein levels of p-JNK, JNK, Beclin-1, p62, and *β*-actin by Western blotting. (d) Levels of p-JNK relative to those of JNK. Levels of (e) Beclin-1 and (f) p62 relative to those of *β*-actin.

Recent evidence has shown that JNK is involved in autophagy [24]. HSPB1 was previously identified as a crucial molecule in the P38 MAPK signaling pathway [25]. Therefore, we attempted to investigate whether HSPB1 mediates autophagy through activation of the JNK pathway. First, we validated the effect of HSPB1 overexpression or silencing on JNK levels. As expected, HSPB1 overexpression markedly increased the expression of p-JNK, while downregulation of HSPB1 expression attenuated JNK phosphorylation. However, no significant differences in total JNK levels were found (Figures 4(a) and 4(b)). We then used a JNK agonist (anisomycin) and inhibitor (SP600125) to verify the role of HSPB1 in regulating the JNK/Beclin-1 pathway in VSMCs. We observed that overexpression of HSPB1 could significantly reverse the suppressive effect of SP600125 on JNK phosphorylation and that silencing of HSPB1 promoted the suppressive effect of SP600125 on p-JNK in VSMCs stimulated by ox-LDL (Figures 4(c)–4(f)). Importantly, we found that HSPB1 siRNA reversed the anisomycin-induced increase in the expression of Beclin-1 and decrease in the expression of p62 in ox-LDL-treated cells. In contrast, upregulation of HSPB1 expression markedly attenuated the increase in p62 expression and the decrease in Beclin-1 expression after SP600125 treatment (Figures 4(c)–4(f)). Taken together, these results demonstrate that activation of the JNK/Beclin-1 pathway is vital for HSPB1-mediated protective autophagy in VSMCs.

## 4. Discussion

**Figure 5:**
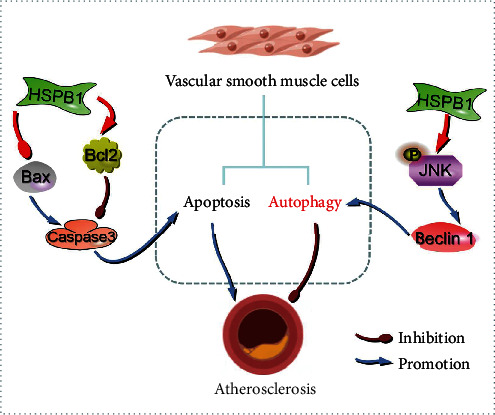
Schematic illustrating the working principle of HSPB1-related signaling in VSMCs. We propose that HSPB1 maintains the phosphorylation state of JNK, thereby activating Beclin-1-dependent autophagy associated with atherosclerosis. Additionally, HSPB1 plays an important role in VSMC apoptosis.

ASO is an ischemic disease of the lower limbs that is primarily caused by atherosclerotic changes in the lower limb arteries, resulting in gradual arterial stenosis and occlusion [26]. An increasing number of studies have confirmed that the inflammatory response plays an important role in the development and progression of ASO. Importantly, excessive proliferation of VSMCs is the main factor that promotes the formation of atherosclerotic plaques and aggravates the development of atherosclerosis [27]. The biological function of HSPB1 is to protect cells from various stressors, such as free radicals, heat, ischemia, and toxic substances, in the environment. In addition, HSPB1 is involved in signal transduction and regulation during cell proliferation, differentiation, and apoptosis [28]. However, very little research has focused on the role of HSPB1 in ASO. In the present study, we found that HSPB1 downregulates the expression of cleaved caspase-3 by decreasing the level of Bax and increasing the expression of Bcl-2 in VSMCs. Importantly, our results revealed that HSPB1 plays an important role in resisting ox-LDL-induced autophagy through the JNK/Beclin-1 signaling pathway (Figure 5). Our research suggests that HSPB1 can alleviate ox-LDL-induced injury in VSMCs by inhibiting apoptosis and promoting autophagy.

In recent years, the physiological function of HSPB1 and its relationship with clinical diseases have received increasing attention from researchers. Studies have shown that the level of HSPB1 in atherosclerotic plaques is significantly lower than that in healthy blood vessels; moreover, the plasma levels of HSPB1 are dramatically decreased in patients with carotid atherosclerosis compared with healthy people [29, 30]. In this study, we found that HSPB1 was downregulated in plasma specimens from ASO patients. Importantly, we also found that compared to that in patients with grade I and II ASO, serum HSPB1 levels in patients with grade III and IV ASO were more significantly decreased. These results suggest that a low level of HSPB1 may be a biological marker of ASO.

Imbalance in cholesterol metabolism can promote the generation and progression of AS [31], and apoptosis of VSMCs leads to reduced secretion of extracellular matrix (the main component of the fibrous caps covering plaques). When the fibrous caps of plaques become thinned and degraded, the plaques become unstable, and rupture can occur [32, 33], leading to adverse cardiovascular events. The study found that a decrease in intracellular HSPB1 levels weakened the protective effect of HSPB1 against proteolysis-induced VSMC apoptosis [30]. VSMCs can migrate from the medial membrane to the intima, where they proliferate and secrete extracellular matrix, thus forming a fibrous cap that stabilizes atherosclerotic plaques [34, 35]. Of note, if apoptotic VSMCs cannot be cleared in a timely manner, the inflammatory response will be exacerbated, and atherosclerosis development will be promoted [36]. This study found that HSPB1 expression was decreased and caspase-3 expression was increased in the smooth muscle of damaged blood vessels in ASO patients, and we further demonstrated that HSPB1 inhibited caspase-3-dependent apoptosis of VSMCs by regulating Bcl-2 and Bax.

Autophagy aids cell recovery by degrading damaged intracellular substances and plays an important role in preventing atherosclerosis. Autophagy is gradually impaired with the development of atherosclerosis. An increasing number of studies have shown that dysfunctional autophagy is closely related to atherosclerosis. p62 is involved in the autophagy process, and an increase in its level often indicates autophagy dysfunction. p62 aggregation is observed in atherosclerotic plaques [37], and as the plaque burden and age increase, the level of p62 in plaques further increases [38]. Our studies also found that the expression of p62 was increased in the intima of damaged vessels in patients with ASO. Notably, a study revealed that loss of GAB1 promotes VEC autophagy, which is associated with ASO [39]. The above studies suggest that autophagy dysfunction plays a certain role in regulating the pathophysiology of atherosclerosis. Recently, some studies suggested that HSPB1 promotes autophagy [16, 17, 40]. Researchers have found that P2RX7 regulates autophagy by fine-tuning HSPB1 expression in astrocytes [41]. In line with this, our study demonstrated that HSPB1 increased the phosphorylation of JNK, promoted Beclin-1-dependent autophagy, and ameliorated ox-LDL-induced injury to VSMCs.

In this study, we demonstrated that JNK and Beclin-1 inhibitors significantly antagonized VSMC autophagy, suggesting that JNK and Beclin-1 may not be essential for HSPB1-induced cell autophagy. We also cannot rule out the possibility that HSPB1 disrupts the Beclin-1/Bax complex by interacting with the activator of JNK, thus promoting autophagy and inhibiting apoptosis. In addition, there are some limitations to this experiment. The role of HSPB1 was studied in VSMCs but not in animal models of ASO. Our next study will focus on the mechanism by which HSPB1 regulates host autophagy and apoptosis in rat models.

However, taken together, our results showed that HSPB1 expression is decreased in patients with ASO. Furthermore, we found that HSPB1 plays a negative role in promoting apoptosis and a positive role in promoting autophagy in VSMCs. This finding suggests a new mechanism underlying the development of ASO pathogenesis, providing a new target for future drug development.

## 5. Conclusion

The present study preliminarily demonstrated that HSPB1 plays a role in inhibiting apoptosis and promoting autophagy in VSMCs, thus maintaining the normal morphology and function of the cells. HSPB1 may be an important molecule for reducing the occurrence and development of atherosclerosis, and it is also an important molecular target for the treatment of ASO.

## Figures and Tables

**Table 1:** 

Characteristics	Fontaine's classification				*p* value
	I (*n* = 39)	II (*n* = 29)	III (*n* = 10)	IV (*n* = 13)	
Female (*n*)	26 (66.67)	19 (65.51)	4 (40.0)	5 (38.46)	0.156
Age (mean ± SD years)	58.36 ± 10.89	67.62 ± 8.01	66.50 ± 10.18	74.92 ± 9.70	0.321
Smoker, *n* (%)	21 (53.84)	12 (41.38)	6 (60.0)	9 (69.23)	0.369
Drinker, *n* (%)	25 (64.10)	18 (62.07)	7 (70.0)	10 (76.92)	0.796
Hypertension, *n* (%)	36 (92.31)	21 (72.41)	10 (100)	12 (92.31)	0.095
Hyperlipidemia, *n* (%)	22 (56.41)	15 (51.72)	8 (80.0)	11 (84.62)	0.113
Diabetes mellitus, *n* (%)	23 (58.97)	16 (55.17)	9 (90.0)	9 (69.23)	0.226
FPG (mmol/l)	7.57 ± 1.15	8.21 ± 1.68	9.18 ± 1.50^∗^	9.83 ± 1.17^#^	0.001
TC (mmol/l)	5.07 ± 0.19	5.34 ± 0.13	5.79 ± 0.19	6.15 ± 0.16^#^	0.022
TG (mmol/l)	1.88 ± 0.75	2.00 ± 0.78	1.89 ± 0.82	1.97 ± 0.91	0.201
HDL-C (mmol/l)	1.43 ± 0.11	1.39 ± 0.10	1.34 ± 0.12^∗^	1.32 ± 0.11^#^	0.010
LDL-C (mmol/l)	3.38 ± 0.45	3.44 ± 0.47	3.61 ± 0.44	4.23 ± 0.34^#^	<0.0001

The data are presented as the mean ± SD or number (percentage). Smoker: the patient's duration of smoking cessation exceeded three months, but the patient had smoked at least five cigarettes a day in the past two years or was a nonsmoker within three months. Drinker: drinks more than 4 drinks a day. FPG: fasting plasma glucose; TC: total cholesterol; TG: triglyceride; HDL-C: high-density lipoprotein cholesterol; LDL-C: low-density lipoprotein cholesterol. ∗ vs. grade I, p<0.05; # vs. grade II, p<0.05.

## Data Availability

The data used to support the findings of this study are included within the article.
